# GMATA: An Integrated Software Package for Genome-Scale SSR Mining, Marker Development and Viewing

**DOI:** 10.3389/fpls.2016.01350

**Published:** 2016-09-13

**Authors:** Xuewen Wang, Le Wang

**Affiliations:** ^1^Germplasm Bank of Wild Species in China, Kunming Institute of Botany, Chinese Academy of SciencesKunming, China; ^2^Key Laboratory of Forensic Genetics and Beijing Engineering Research Center of Crime Scene Evidence Examination, Institute of Forensic Science, Ministry of Public SecurityBeijing, China

**Keywords:** SSR software, marker polymorphism and transferability, statistical graph, Gbrowser display, grass genome SSR pattern

## Abstract

Simple sequence repeats (SSRs), also referred to as microsatellites, are highly variable tandem DNAs that are widely used as genetic markers. The increasing availability of whole-genome and transcript sequences provides information resources for SSR marker development. However, efficient software is required to efficiently identify and display SSR information along with other gene features at a genome scale. We developed novel software package Genome-wide Microsatellite Analyzing Tool Package (GMATA) integrating SSR mining, statistical analysis and plotting, marker design, polymorphism screening and marker transferability, and enabled simultaneously display SSR markers with other genome features. GMATA applies novel strategies for SSR analysis and primer design in large genomes, which allows GMATA to perform faster calculation and provides more accurate results than existing tools. Our package is also capable of processing DNA sequences of any size on a standard computer. GMATA is user friendly, only requires mouse clicks or types inputs on the command line, and is executable in multiple computing platforms. We demonstrated the application of GMATA in plants genomes and reveal a novel distribution pattern of SSRs in 15 grass genomes. The most abundant motifs are dimer GA/TC, the A/T monomer and the GCG/CGC trimer, rather than the rich G/C content in DNA sequence. We also revealed that SSR count is a linear to the chromosome length in fully assembled grass genomes. GMATA represents a powerful application tool that facilitates genomic sequence analyses. GAMTA is freely available at http://sourceforge.net/projects/gmata/?source=navbar.

## Introduction

Simple sequence repeats (SSRs), also called microsatellites (repeat unit length ≤6 bp) or minisatellites (unit length ≥6 bp), are relatively short tandem repeats (STRs) of DNA (Ellegren, 2004; Sharma, 2007) that are widely distributed throughout whole genomic sequences. In eukaryotic species, approximately 10 to 20% of genes and promoters contain microsatellites (Gout et al., 2013). The length of an SSR shows extensive intraspecies and interspecies variations, which is primarily because of the high rates of DNA replication errors within SSRs (Ellegren, 2004; Klintschar et al., 2004; Forster et al., 2015). Thus, SSRs are widely used as markers, although the availability of single nucleotide polymorphisms (SNPs) as a marker resource has increased with advancements in next-generation sequencing technology (Davey et al., 2011). However, SSR markers are often the first choice in certain applications. In forensics, for example, only a few SSR markers are informative enough to identify DNA differences between human individuals. Breeders prefer SSR markers because they are much easier to use in molecular labs than SNPs, which require additional expensive equipment. The traditional method of SSR marker development is time consuming because an SSR sequence must be obtained, a primer pair that flanks the SSR must be designed, an experimental PCR must be conducted, and the marker's polymorphism must be scored after separation by electrophoresis.

Bioinformatic tools have been developed for automated SSR discovery and/or marker development. These tools, such as Tandem Repeat Finder (TRF) (Benson, 1999), MISA (Thiel et al., 2003), and SciRoko (Kofler et al., 2007), conduct SSR mining, whereas other tools, such as SSRLocator (da Maia et al., 2008) and SSRPoly (Duran et al., 2013), also design primers. Thus, these tools greatly speed up SSR analyses and marker design for certain species, e.g., *Setaria* (Pandey et al., 2013) and cotton (Wang et al., 2015). With advancements in next-generation sequence technologies, the ever-increasing availability of whole-genome and transcript sequences provides considerable data resources for SSR marker development. To identify and fully utilize the SSRs from these available sequences requires high-efficiency bioinformatic tools. However, the tools mentioned above are limited and do not meet the requirements of the genome era. Currently available SSR tools have several major limitations. First, the tools have insufficient sequence processing capabilities to process large genome sequences, which has been summarized by Sharma (2007). These tools usually require a long run time or lack of capability when analyzing whole-genome sequences. Second, limited statistical analyses are provided by the available software, such as TROLL (Castelo et al., 2002). In addition, certain tools display platform dependence, such as SSRLocator (da Maia et al., 2008), which only runs on Microsoft Windows, a system that is known to have a poor capacity for large data set analyses. Most command tools do not provide a graphical interface [i.e., MISA (Thiel et al., 2003)]; therefore, the use of these systems may be problematic for non-bioinformaticians. Third, the available software does not have the capacity for efficient marker design, and most of the tools that perform marker design, such as SSRPoly (Duran et al., 2013) and CandiSSR (Xia et al., 2016), are time-consuming pipelines that merely integrate with previous primer design software.

Despite the large number of available tools, none possesses multiple functions for the identification of SSRs, characterization of their distribution, design of SSR markers, and presentation of SSR information on a genome scale. Our previous software GMATo was developed for fast and accurate SSR discovery and statistical analyses at the genome level to overcome the limitations and challenges mentioned above (Wang et al., 2013). The increasing needs from users inspired us to produce a novel software package, GMATA, which provides new strategies and complete solutions for fast SSR analyses, marker development, polymorphism screening by mapping and graphical display of results in a genome browser with other genic features. This software also provides high-quality statistical graphics for direct incorporation in publications. GMATA is an advanced, powerful application tool with multiple functions for SSR and marker analysis on a whole-genome scale.

## Materials and methods

### GMATA strategy for SSR identification

**Figure 1:**
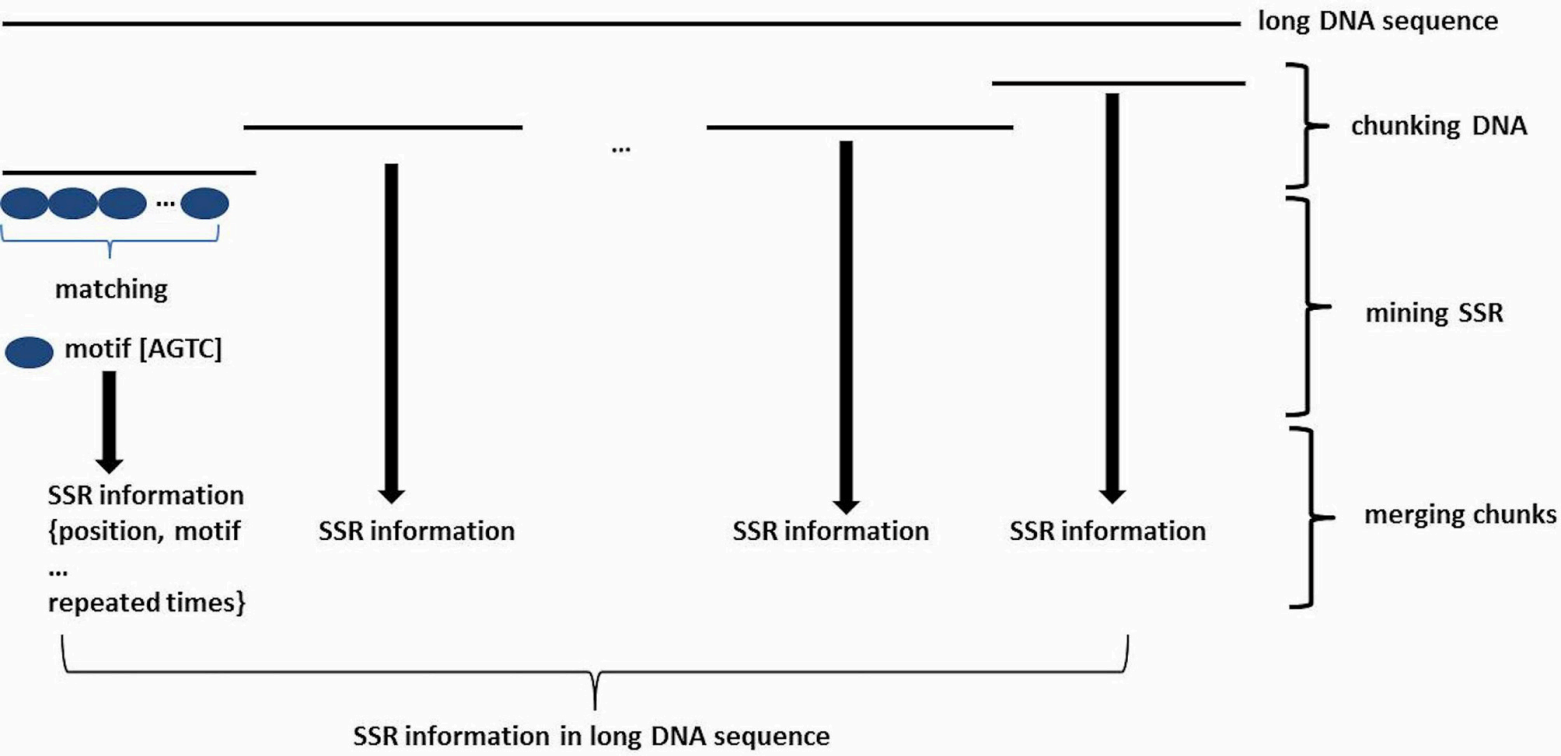
**Schematic diagram showing the SSR mining strategy in GMATA for a long DNA sequence**. Prior to SSR mining, a long DNA sequence is chunked into DNA fragments of an appropriate length with an overlapping sequence at each end. After SSR mining within each DNA chunk, SSR loci information from each segment is combined to generate the final SSR information in the original long DNA sequence.

To mine SSRs efficiently from a long DNA sequence, we applied a strategy of chunking a long DNA sequence (default at >2 Mb) to an appropriate length to increase mining speeds. A short overlapping region (default 20 bp) at the end of each chunk provides for accurate SSR identification at the end of the sequence (Figure 1). After mining, the SSR information is then reconstructed back to the level of the long DNA sequence. To identify SSRs, basic SSR units consisting of nucleotides A, T, G, and C are dynamically computed as a motif library at a user-controlled length using meta-characters and a regular expression patterning algorithm from the computing language Perl version 5. Then, repeated motifs are greedily researched using Perl's pattern matching algorithm. The returned values are used to generate SSR loci information. The SSR mining module in GMATA allows the user to adjust certain parameters, including the motif length range and the minimum times the unit is repeated, and it also provides an option to output highlighted microsatellites in the target DNA sequence. The unit length can be set to any range, not limited to a maximal length of 6 or 10 bp which is set in existing SSR mining tools.

### SSR statistical classification and graphics plotting

The statistical classification and summarization are sorted into five classes as follows:

- motif type based on the unit length; the motif type (MT) is measured by the length (L) of the repeated motif unit,
  $$\text{MT}=\sum_{i\;=\;1}^{i}L\left(\text{i}\right)$$
- motif composition, which is composed of any combination of the nucleotides A, G, T, and C;
- grouped motif units, which are grouped if two motifs are complementary to each other; if the DNA orientation on the chromosome is unknown, these complementary motifs are considered the same, and the two motifs are grouped together;
- SSR distribution over each chromosome, which represents the frequency of SSRs per million bases (Mb) of DNA as calculated by the formula
  frequency=100000*Σ (SSR)/(Lenght)
  where “SSR” represents the SSR count and “Length” represents the length of the source DNA;
- distribution of SSR length (SL), which is calculated by
  $$SL=\sum_{i\;=\;1}^{i}l,$$

where “l” represents the SSR count at a particular SSR length.

Each classification result provides a class name, count and percentage in descending rank. Statistical graphics are also plotted as multiple chart types (e.g., distribution frequencies are plotted as both a pie chart and a bar chart).

### Improved primer design and marker generation

To efficiently design primers for each SSR locus in a long genomic sequence, we used a new strategy to improve the speed of the primer design. First, all of the SSR loci and genome sequences are indexed. Then, a short flanking sequence to either side of each SSR locus is extracted at a user-controlled length (default 400 bp) instead of using the full-length DNA sequence, which is performed in other reported tools used for primer design. Primers are designed using the Primer3 algorithm from the flanking sequences, and one primer is located on each side of an SSR. Users can configure parameters such as a range of amplicon sizes (default 100–400 bp) and annealing temperatures (default 60°C). Primer pairs are clustered to a unique pair if 100% identical among all the designed primers, and then a unique marker ID is assigned. The primer information is saved in “.mk” and “.sts” files in the NCBI's STS format, containing the marker name, its primer sequences and amplicon size, ready for primer ordering.

### Polymorphism scoring by electronically mapping markers

To determine the polymorphism of any designed markers in any given DNA sequence, simulated marker mapping (e-mapping) is performed based on a forward e-PCR algorithm (Schuler, 1997). A marker file (.sts) and a DNA sequence file should be used as input in this e-mapping module. The e-PCR parameters can be derived from the default values or set by the user. An e-PCR report file (.amp) is generated. To calculate the polymorphism, all of the amplified fragments from each marker in a given genome are scored by size. Then, the polymorphism information (suffix.frg) and mapping information (suffix.emap) are produced. A summary of the e-mapping results is also produced (.sat4) that provides the distribution of alleles for all markers, total number of sequences with marker(s) mapped, total number of mapped markers, total amplicons, and the average number of amplicons per mapped marker.

### Implementation

**Figure 2:**
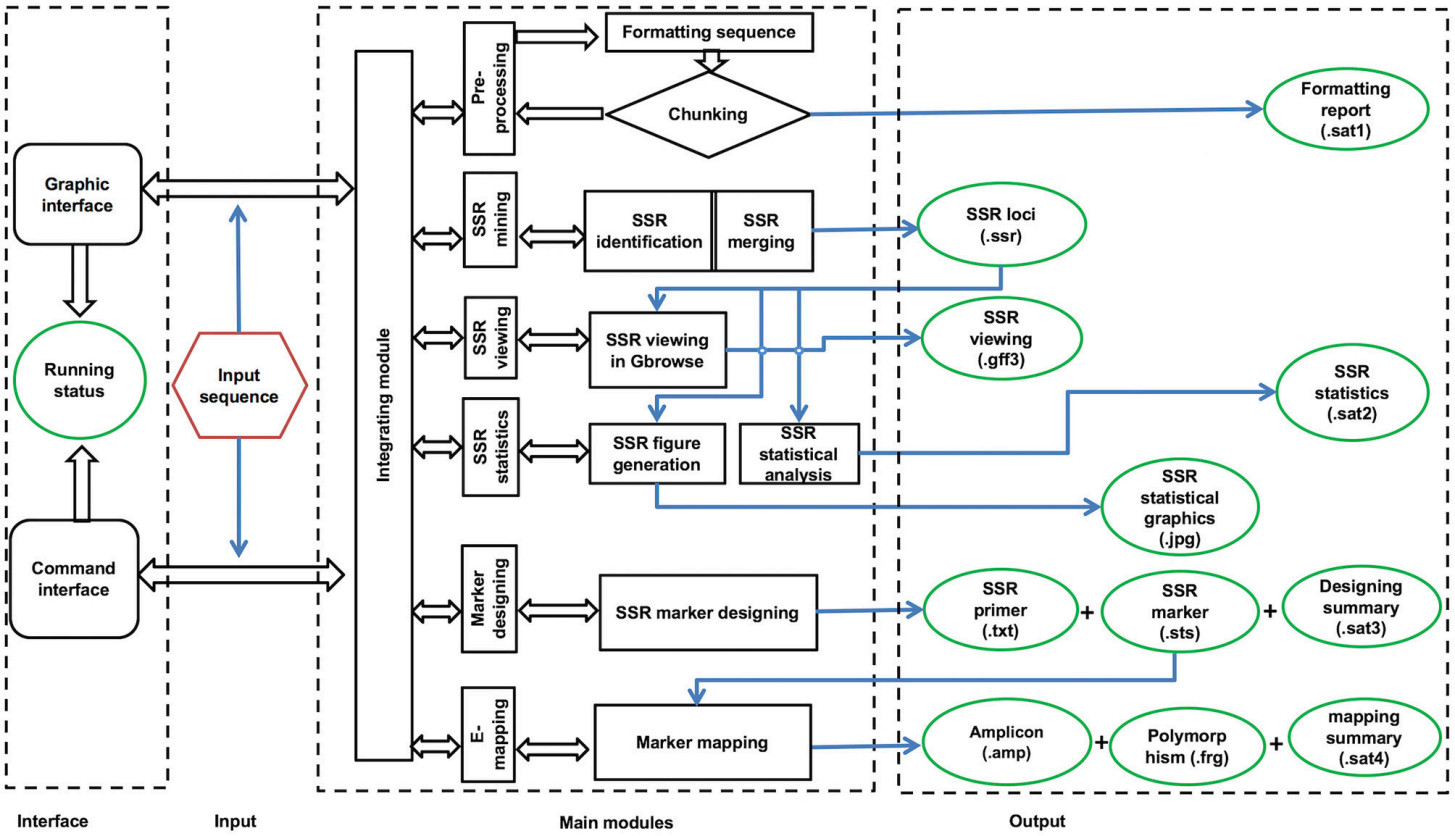
**Flowchart of the software GMATA**.

The workflow of the GMATA software consists of six modules, including DNA pre-processing, SSR mining, SSR viewing, SSR statistics, marker design, and electronic mapping of the marker (Figure 2). Each module provides full and easy parameter control at the user's preference or with the default settings.

**Figure 3:**
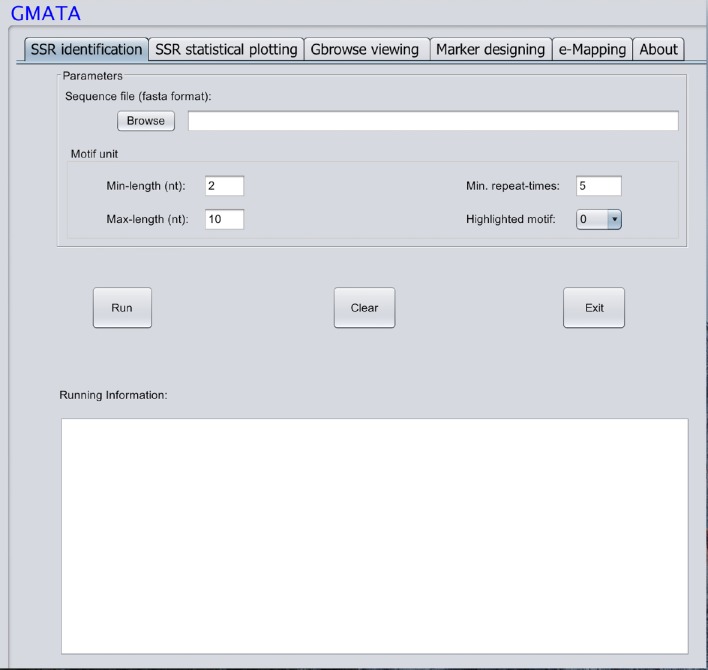
**The graphical interface of the GMATA software**.

GMATA was packed into a package that integrates all modules, which work together seamlessly regardless of whether the modules were written in Perl (64 bit, version 5), R (version 3.0) or Java (version 7). Each module can also be invoked independently. Perl scripts are used to perform formatting and chunking raw DNA sequences, identifying microsatellites, designing primers, and markers and e-mapping markers. Java scripts are used for developing the graphical user interface (GUI), which internally calls Perl scripts. Both Perl and R scripts are used to perform statistical analyses and plot statistical graphics. The GMATA software is compatible with multiple platforms and has been tested and shown to work in Linux, Mac OS 10 and Windows 7, 8, or 10 systems. Either a graphical GUI or command line can be invoked to run the GMATA software. The GMATA software provides user-friendly graphical and command line interfaces (Figure 3). The command line can be easily integrated into any automated pipeline. A one-step utility has also been developed for automatically running all modules or any combination of modules by configure a setting file.

Only one input file of DNA sequence(s) in a flexible FASTA format is required for SSR mining, which is performed via mouse clicks in the graphical interface or typed via the command line. A second sequence file in the FASTA format may be required if the user wants to transfer the designed markers to other DNA sequences. GMATA can also receive DNA sequences or next generation sequence file in fastaq format if using our utility provided.

### Experimental validation

We mined SSR and designed markers from seven *Nicotiana* genomes from the links in the previous review (Wang and Bennetzen, 2015). To test whether the newly designed SSR markers are amplifiable, an ePCR analysis was conducted for several *Nicotiana* species and varieties. Genomic DNA was extracted from the leaves of tobacco accessions *N. sylvestris, N. tomentosiformis, N. tabacum* HD, *N. tabacum* K326, and *N. benthamiana* and five *N. tabacum* varieties (Yunyan-85, Yunyan-97, Zhongyan-100, KRK26, and CB-1) by using the DNeasy Plant Mini Kit (Qiagen Inc., Valencia, CA, USA, Cat. No. 69104). DNA was diluted to 30–50 ng/μl in water before use.

The tailed forward primer was synthesized with the tail sequence CGTTGTAAAACGACGGCCAGT added to the 5′ end of each NIX forward primer. The PCR analysis was performed in 20 μl volumes containing 30–50 ng DNA, 0.13 μM tailed forward primer, 0.27 μM 5′ FAM, or 5′ HEX florescent-labeled primer CGTTGTAAAACGACGGCCAGT, 0.53 μM reverse primer, and 10 μl Phusion Hot Start Flex 2 × Master Mix (New England BioLabs, Ipswich, MA, USA, Cat. No. M0536S). PCR amplification and amplicon scoring were conducted according to our previous description for UGSW markers (Serba et al., 2013).

## Results

### Performance of the GMATA software

To compare the performance of the GMATA software, two commonly used tools, SSRLocator (da Maia et al., 2008), and MISA (Thiel et al., 2003), were used as controls for the whole-genome SSR identification and primer design using the 500-Mb whole-genome sequence from *Setaria italica* (Bennetzen et al., 2012) and 2-Gb sequence from *Zea mays* (Schnable et al., 2009). Only the GMATA and MISA software packages were able to mine the SSRs in the large *Z. mays* genome although all of the assessed tools could mine SSRs in the small *Setaria* genome. GMATA ran much faster than the other two tools on all three major computing platforms (Windows, Linux, and Mac OS; Supplementary Table 2). Much less computing memory was used by GMATA, which allowed GMATA to mine SSRs from the whole-genome sequence on a common computer (Supplementary Table 2). GMATA, SSRLocator, and MISA identified a total number of 46,739, 46,625, and 46,782 SSR loci in *S. italica*, respectively. Further manual comparisons of the SSR loci revealed that the extra loci reported by MISA were redundantly overlapped, suggesting more accurate, and sensitive SSR mining from GMATA than the other two tools (Supplementary Table 2). Compared with other software, the GMATA software offers novel functions that include super-fast primers and marker design from whole-genome sequences. For GMATA, the average time needed to design primers and markers from whole-genome SSR loci was approximately 3 and 19 min in *Setaria* and maize, respectively. However, the MISA and SSRLocator software cannot easily design primers from whole genomic sequences (Supplementary Table 2). We also successfully applied GMATA for SSR identification in the massive 22.1-Gb genome sequence of *Pinus taeda* (http://dendrome.ucdavis.edu/treegenes) (Neale et al., 2014) within 2.5 h on a common laptop that only has 2.8 Gb of memory, which is currently impossible for the other available tools.

### Output of the software GMATA

#### SSR loci results

After running the SSR identification by GMATA on a genomic DNA sequence, a SSR loci information file (.ssr) can be generated providing SSR starting position, ending position, repetitions, and motif (Table 1). A summary file (.sat1) is also generated for the total number and total length of the input sequences.

**Table 1:** **Output of SSRs identified by GMATA from the ***Setaria*** genome**.

**Name**	**Seq_Len**	**StartPos**	**EndPos**	**Repetitions**	**Motif**
>scaffold_1	42145699	2693	2704	6	TA
>scaffold_1	42145699	3225	3236	6	AG
>scaffold_1	42145699	10151	10160	5	TA
>scaffold_1	42145699	30866	30875	5	TA
…					
>scaffold_9	58970518	58865464	58865478	5	CAC
>scaffold_9	58970518	58898017	58898062	23	GT
>scaffold_9	58970518	58944715	58944726	6	GT
>scaffold_9	58970518	58953059	58953070	6	GT
>scaffold_9	58970518	58953641	58953654	7	TG
>scaffold_9	58970518	58968425	58968434	5	TA
>scaffold_9	58970518	58968954	58968965	6	AG

Example data showing the output results of SSR loci information by GMATA in the Setaria genome, which was downloaded from Phytozome (assembly version 164). The parameters were set to the GMATA defaults.

#### SSR statistical analysis and graphic plotting

**Figure 4:**
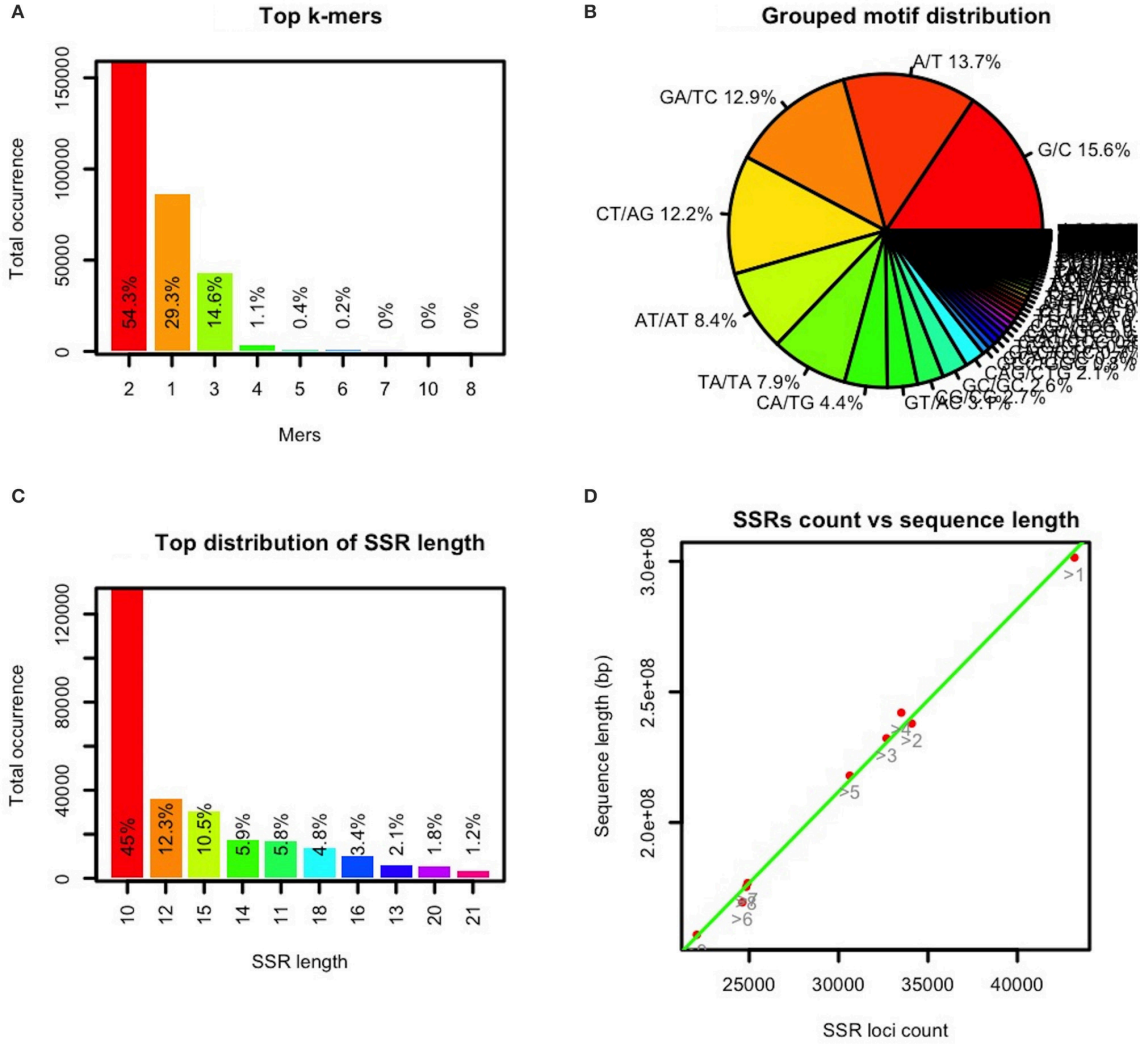
**Selected statistical graphics by GMATA for the maize genome**. SSRs (monomers with a minimum of 12 repeats and unit length of 2–10 bp) in the maize genome sequence were analyzed in GMATA using the default settings. Statistical plotting was conducted using the default settings. **(A)** shows the mer or length distribution of the repeated motif, **(B)** shows the distribution of the repeated motif nucleotides, **(C)** shows the length distribution of the SSRs, and **(D)** shows the distribution of the SSR counts vs. the length of each chromosome (bp).

In GMATA, a SSR statistical module uses the SSR loci file (.ssr) to generate the statistical “.sat2” file and perform graphical plotting of each statistic. The file provides statistical data for the five genomic classifications: motif type, motif composition, grouped complementary motifs, SSR distribution for each DNA molecule or chromosome, and SSR length (Supplementary Data S2). GMATA calculates the SSR distribution on a single DNA molecule, providing clues to SSR distribution on each chromosome in a well-assembled genome (Supplementary Data S2). The GMATA plotting module generates statistical graphics configurable by the user (Figure 4). The graphics clearly show the distribution of the repeated motif's mers and nucleotides, SSR length (Figures 4A–C) and SSR occurrence at a chromosome level.

#### Genome-wide SSR marker design

The primer-design module uses the SSR loci and their original DNA sequences to design the primers. The output is the primer information in the NCBI “.sts” format (Table 2) and also a much detailed primer information in the template DNA sequence (Supplementary Data S3).

**Table 2:** **Example data showing the markers designed by GMATA**.

**Marker ID**	**Left_primer_sequence**	**Right_primer_sequence**	**Product_size**	**Repeated motif**
>MK1	GTCATTTGCCGACCAAACAT	CGGGAGAGGTAAAGGGAAGA	338	AAATAA
>MK2	TGATGCACCCTTTGTGGTTA	CAAAATTGGCATCAAACACG	149	AT
>MK3	TGGCTAGATTCAAATCGTTCAG	CTAAGCACTACCCCGAGCAT	214	TG

Example data showing the output file (.sts) of the SSR marker designed from the Nicotiana genome sequence using GMATA's default settings.

#### Marker mapping results and polymorphisms

To develop genome-wide genetic markers, the polymorphism information of a marker, such as the number and size of the alleles, is extremely useful. In GMATA, e-mapping of a marker is conducted through a simulated *in silico* PCR using the e-PCR algorithm (Schuler, 1997) to generate the amplicon and then calculate the allele's size. Two results will be generated: (i) the allele information for each marker and (ii) the mapped markers on each DNA sequence/chromosome. We applied GMATA to design and map SSR markers from the pooled genomic sequences of seven *Nicotiana* species/varieties. A batch of polymorphic markers were discovered (Table 3). The user also can use GMATA to map and screen polymorphism information for any pre-existing marker.

**Table 3:** **Examples showing the polymorphisms of markers analyzed by GMATA**.

**Marker_ID**	***N. ben***	***N. syl***	***N. tom***	***N. oto***	***N. tab* TN90**	***N. tab* K326**	***N. tab* BX**
>NIX1	567	NA	338	NA	338	338	338
>NIX101397	149	NA	NA	NA	151	151	151
>NIX101398	202	200	205	204	200 + 204	200 + 204	200 + 204
>NIX118301	NA	NA	313	2531 + 284	320	320	320

The data showed the size of the simulated PCR amplicons of each marker. SSR markers designed from mixed genomic sequences of seven Nicotiana species/varieties were mapped by GMATA (using the default settings) to each genome to determine the allele polymorphisms. N. ben, N. syl, N. tom, N. oto, N. tab TN90, N. tab K326, and N. tab BX represent Nicotiana accessions. NA indicates no amplicon was obtained for the marker.

#### Visualization of SSR with genome features

**Figure 5:**
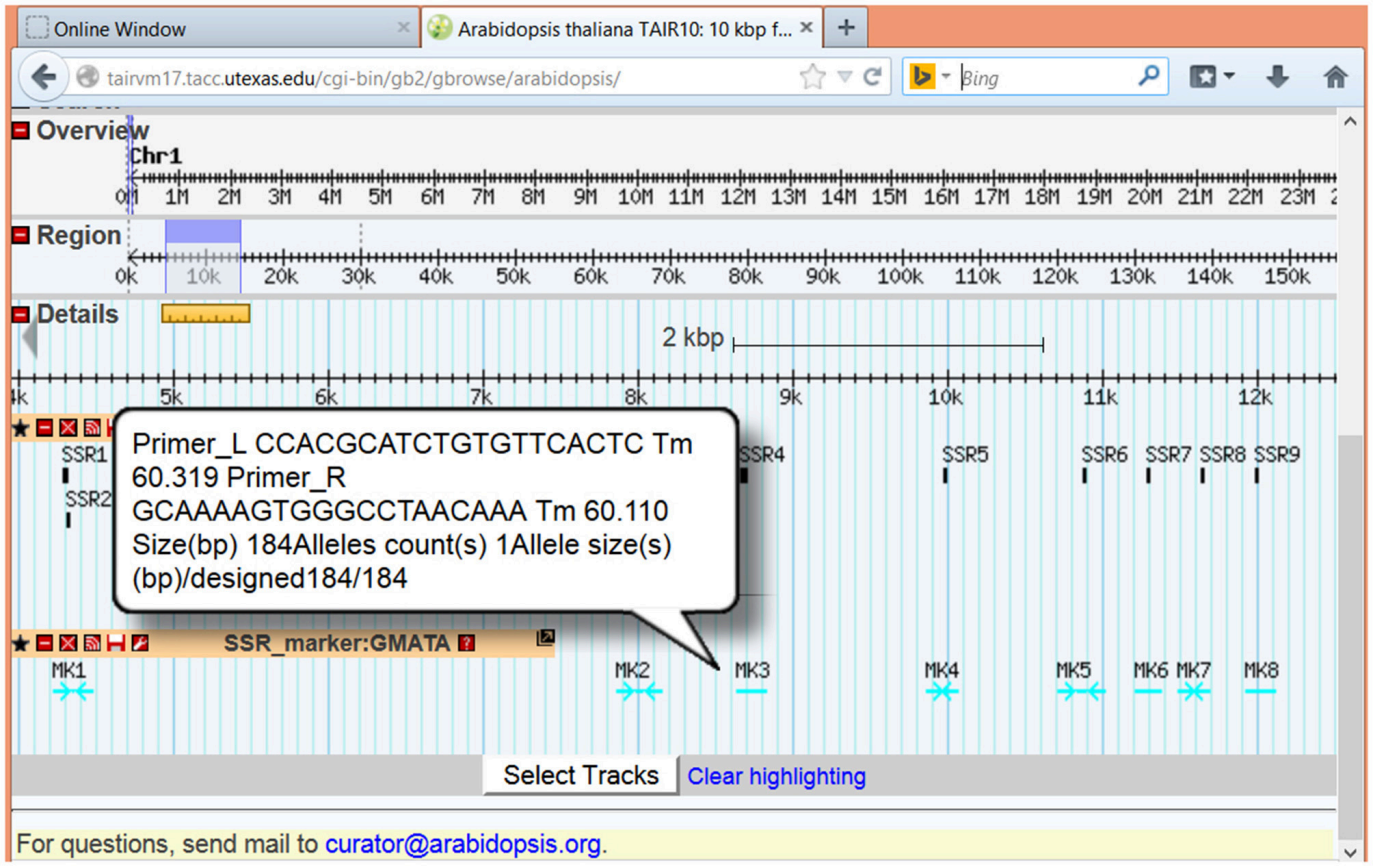
**Images showing the display function of GMATA**. SSRs (unit length 2–6 bp) from the *Arabidopsis* genome sequence were analyzed in GMATA using the default setting. The output from *Arabidopsis* with the suffix.gff3 was uploaded to the online Gbrowse browser at TAIR and displayed with other genome features. Both SSR loci and SSR marker information are displayed for the *Arabidopsis* genome sequence.

To view the SSR loci and marker together with other genome information, GMATA can generate a genome browser-supported feature file (.gbf or.gff3) for directly input and display in genome browsers online or locally, such as GenomeView (Abeel et al., 2012) or Gbrowse (Stein et al., 2002). Here, we show examples of the graphical presentation of SSR loci at online genome databases TAIR (www.arabidopsis.org) (Figure 5) and phytozome (www.phytozome.com) (Supplementary Figure 1). Detailed information on each SSR loci or maker can be viewed after pointing or clicking on the marker.

### A novel SSR distribution pattern in grass genomes revealed by GMATA

The grass family (*Poaceae*) consists of the most important food crops, including rice and wheat, and SSR information from these plants can be used for crop improvement. However, a systematic knowledge of SSRs across grass genomes is largely unknown. We analyzed the SSRs over all 15 *Poaceae* genomic assemblies available to date (Supplementary Table 1) using GMATA at equivalent SSR mining settings as previously reported (Ling et al., 2013), and our results revealed a novel SSR distribution pattern in the grass genomes (Supplementary Table 3). In 11 genomes, the dinucleotide motifs (dimers) are the most abundant, followed by monomer motifs and then trinucleotide (trimers) motifs. The remaining 4 genomes (*Lolium perenne, Brachypodium distachyon, S. italic*, and *Oryza glaberrima*) presented more monomer motifs than dimers followed by trimers. Although the grass genomes are known to be G/C rich, we found that the SSR motifs in the grasses did not display similar sequence enrichment. Most of the grass genomes (11 of 15) were rich in A/T monomers, whereas the remaining four genomes were rich in G/C monomers. For the dimers, GA/TC was the most abundant motif in 11 of 15 genomes, and it was followed by the AT/AT motif in three genomes. Among the trimers, GCG/CGC was the most abundant, and it was followed by CTC/GAG in several grasses. Most of the SSRs were located in intergenic regions, whereas only a few SSRs (7–32%) were located in genic regions. However, 42% of the SSRs in the assembly of hexaploid wheat were concentrated in genic regions, which may have been related to the current assembly being primarily obtained from low-copy sequence gene regions. A comparison of the SSR density (SSR count per megabase of sequence) revealed that the SSR density was higher in genic regions than intergenic regions. Long SSRs, which should be highly polymorphic, were 1–3% higher in the genic regions than all other genomic regions (Supplementary Table 3).

To date, the genomic sequence of the five listed grass genomes, *B. distachyon, Oryza sativa, S. italica*, sorghum, and *Zea mays* have been assembled at the chromosome level. After analyzing the SSRs in these genomes using GMATA, the occurrence of SSRs on the chromosome was found to be linear to the length of the chromosomes in the genomes (Figure 4D).

### Experimental validation of the markers designed by GMATA

**Figure 6:**
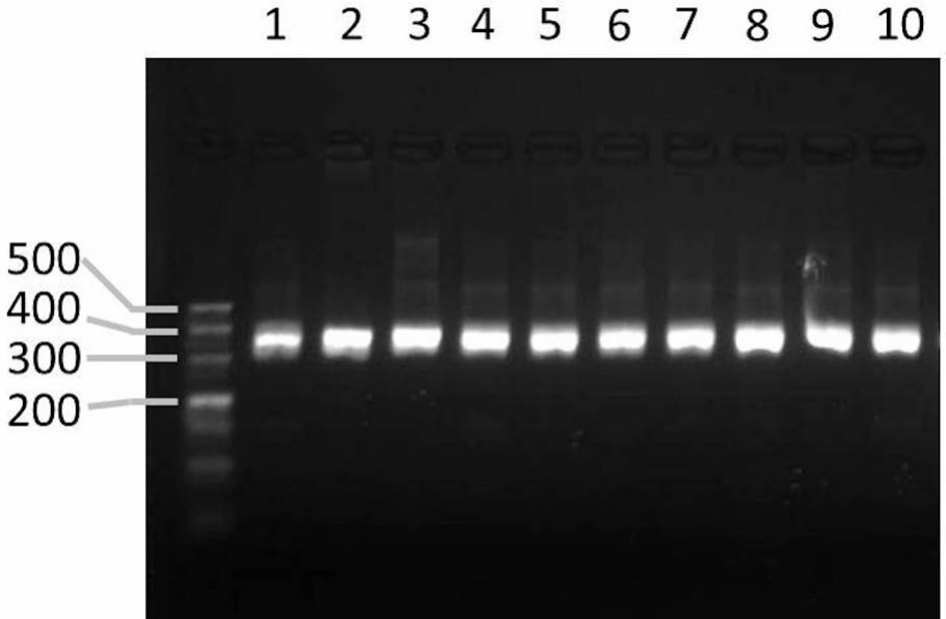
**Agarose gel image showing the PCR products amplified from markers designed by GMATA**. Lanes 1–10 represent the PCR amplicons from the DNA of tobacco species or varieties *N. sylvestris, N. tomentosiformis, N. tabacum* HD, *N. tabacum* K326, and *N. benthamiana* and *N. tabacum* varieties Yunyan-85, Yunyan-97, Zhongyan-100, KRK26 and CB-1.

To validate the markers designed by the GMATA software, markers were designed from the whole genomic sequences of seven *Nicotiana* species. Among these markers, 24 were used for PCR experimental validation among a tested panel of 10 *Nicotiana* species or varieties. All of the markers produced amplicons within the expected size range (Figure 6).

## Discussion

The increasing number of available whole-genome and transcript sequences provide considerable data resources for SSR mining and SSR marker applications for research and genetic improvements. Currently, the bioinformatics tools for SSRs have fallen into two classes. Class I tools are focused on SSR identification and only provide limited statistical support [e.g., Tandem Repeat Finder or TRF (Benson, 1999), MISA (Thiel et al., 2003), TROLL (Castelo et al., 2002) and SciRoko Kofler et al., 2007]. Class II tools are focused on SSR primer design, and most of these programs are pipeline integrating tools from class I combined with a primer designing tool [e.g., SSRLocator da Maia et al., 2008] and pipelines [e.g., SSRPoly (Duran et al., 2013) and CandiSSR (Xia et al., 2016)]. However, all of the tools are limited in their ability to analyze SSRs and design primers from large sequences. The major challenge is the efficient analysis of SSRs, the design of SSR markers from big data, and the integration of SSRs with other genome features to build an SSR network. GMATA software fills this gap. The large capacity and fast processing of GMATA were derived from our novel strategy of chunking long DNA sequences into short DNA segments. A short DNA segment is much easier to manipulate and uses less computing resources; therefore, SSR mining is much faster. Our GMATA software can theoretically mine SSRs from DNA of unlimited length. The caveat is that no significantly improved speed on short DNA sequences. To date, GMATA is the fastest reported software for SSR identification over a large genomic sequence.

GMATA is the first tool that generates results that enable viewing SSR loci and SSR marker information along with other genome features in a genome browser. The genome browser tools are widely used to graphically integrate data containing whole-genome sequences, gene models, SNPs, gene expression, and other features. GMATA's .gff3 and .gbf output formats can be directly imported into genome browser tools, such as GenomeView (Abeel et al., 2012) and to local computers or Gbrowse (Stein et al., 2002) for use in online genome databases, such as Ensembl (http://useast.ensembl.org/index.html), TAIR (www.arabidopsis.org), Phytozome (https://phytozome.jgi.doe.gov/pz/portal.html), and the human genome database at UCSC (http://genome.ucsc.edu). This feature enables a connection between SSRs and other genome features by clicking, sliding, or zooming in the genome browser. To achieve these results in GMATA, only a few clicks by the user are required. A one-step utility for all of the functions in GMATA is also provided to allow the package to be easily integrated into any pipeline. Therefore, GMATA facilitates analyses by all users, including those without a background in genomics and bioinformatics, and allows them to link SSRs with genome features through a network.

GMATA offers more comprehensive functioning for the statistical analysis of SSRs compared with available software/tools, which provide less or no statistical information about SSR distribution. The SciRoko tool (Kofler et al., 2007) has a statistical analysis function, but GMATA provides a greater number of statistical functions at the genome level. The distribution includes five aspects of microsatellites and all of the distributions are presented in detailed tables and graphics. GMATA is also the first software package to generate multiple statistical graphics for SSRs, and the high-quality statistical graphics can be directly incorporated into articles for publication. The statistical graphics clearly display an overview of the SSR distribution. GMATA's new feature of SSR distribution at the whole-genome level enabled our novel discovery of a linear relationship between the SSR number and DNA sequence length in five grass genomes. The SSR distribution provides important clues of SSRs in the genome and throughout evolution.

GMATA software is a rapid and unique genome-wide marker design tool. Current software/tools, such as SSRLocator (da Maia et al., 2008), cannot easily design primers that flank each SSR locus in a large genome sequence because the genome sequence at the chromosome level is too large to be directly used as a template for primer design. For a large genome, primer design can be quite difficult. GMATA software only uses the flanking sequence as a template for designing PCR primers, which reduces the computing memory and speeds up the design process for large data sequences. Furthermore, not all primer pairs are unique at the genome scale because duplicated DNA sequences have arisen during evolution. Therefore, GMATA software generates a unique marker ID for all primer pairs at the genome level if the sequences in the primer pairs are identical. GMATA provides an easier interface to perform marker polymorphism analysis and a more accessible format for the results (e.g., genome-wide allele size and polymorphisms of each marker, marker amplification information on each chromosome and mapping summary). GMATA is also the first software that provides a mapping function to verify the transferability of the markers to other sequences.

The distribution of SSRs was previously reported in several grass genomes, including rice (Zhang et al., 2007), sorghum (Sonah et al., 2011), foxtail millet (Pandey et al., 2013), *Brachypodium* (Sonah et al., 2011), and maize (Xu et al., 2013). However, these investigations were primarily designed for marker development in a single genome, and the settings were varied to identify interesting SSRs. Here, we used GMATA and a setting equivalent to that reported for *Triticum urartu* (Ling et al., 2013) to mine all of the SSRs (unit length 1–10 bp) present in 15 grass genomes. We observed the same exact distribution of SSRs as reported in *T. urartu* (Ling et al., 2013). Grass genome sequences have a high G/C content (Guo et al., 2015), and monocot genomes have high GCG/CGC motifs compared with dicot genomes (Zhao et al., 2014). Does this genome enrichment indicate that SSRs are predominantly of the G/C type in grass genomes? Our investigation indicates that this is not the case. The most abundant motifs in 11 of the 15 analyzed grass genomes were the dimer GA/TC, the A/T monomer, and the GCG/CGC trimer, although exceptions were observed in particular grass genomes. The GCG/CGC-type SSR was predominant among the trimers in the grass genomes but not in all of the SSR mers. We found that in the grass genomes, most of the SSRs (68–93%) were concentrated in intergenic regions vs. gene regions (7–32%), which is consistent with previous findings in the rice *Sativa japonica* (Zhang et al., 2007) and maize (Xu et al., 2013). Interestingly, we revealed that a higher frequency of SSRs (SSR loci number per megabase of nucleotides in gene regions and whole-gene sequence, which indicates a higher SSR frequency) in gene regions than in intergenic regions. This result explains the higher SSR frequency in the low copy number sequence-based assembly of hexaploid wheat (Supplementary Table 3). In all five genomes that were fully assembled at the chromosome level, our investigation revealed an identical distribution relationship, with the SSR count presenting a linear relationship with the chromosome length. Therefore, we identified clear SSR distribution patterns in the grass genomes using GMATA.

## Conclusion

GMATA is the advanced multi-functional software capable of fast SSR mining, marker analyses, statistics calculations, and graphical presentations, especially at the whole-genome level. GMATA enables the display of SSR loci and SSR markers with other genic features; thus, it can easily connect a genomics network with SSR markers. We recommend that GMATA should be routinely used as a one-step tool for SSR analysis and SSR marker development immediately after a new genome sequence assembly is completed. GMATA is an advanced SSR application tool for a broad variety of users in many fields, from genomics to breeding.

## Author contributions

XW designed the study and programmed the software. XW and LW participated in the software evaluation, and wrote the main manuscript text. All authors reviewed the manuscript.

### Conflict of interest statement

The authors declare that the research was conducted in the absence of any commercial or financial relationships that could be construed as a potential conflict of interest.
