# Supplementary material for: GMATA: An Integrated Software Package for Genome-Scale SSR Mining, Marker Development and Viewing
Source: Front Plant Sci. 2016 Sep 13;7:1350. doi: 10.3389/fpls.2016.01350 (PMC5020087; doi:10.3389/fpls.2016.01350)

Supplementary Material

**GMATA: an integrated software package for genome-scale SSR mining, marker development and viewing**

Xuewen Wang^1^*, Le Wang^2^

*** Correspondence:** Corresponding Author: wangxuewen@mail.kib.ac.cn

Contents

[1. Supplementary Data 2](#__RefHeading___Toc454447320)

[Supplementary Data S1 Sources of the DNA sequences 3](#__RefHeading___Toc454447321)

[Supplementary Data S2 Example statistical file output data from GMATA 4](#__RefHeading___Toc454447322)

[Supplementary Data S3 Example marker design output by GMATA 6](#__RefHeading___Toc454447323)

[2. Supplementary Figures and Tables 8](#__RefHeading___Toc454447324)

[2.1 Supplementary Figures 8](#__RefHeading___Toc454447325)

[Supplementary Figure1 The display function of GMATA 9](#__RefHeading___Toc454447326)

[2.2 Supplementary Tables 9](#__RefHeading___Toc454447327)

[Supplementary Table 1 Genomic sequence sources of grass genomes 9](#__RefHeading___Toc454447328)

[Supplementary Table 2 Performance comparison of GMATA, SSRLocator and MISA software on whole genome sequence 11](#__RefHeading___Toc454447329)

[Supplementary Table 3 Distribution of SSR identified by GMATA in fifteen grass genomes 12](#__RefHeading___Toc454447330)

# Supplementary Data

## Sources of the DNA sequences

All DNA sequences used in the study are downloaded from public databases. [*Pinus taeda*](http://www.ncbi.nlm.nih.gov/Taxonomy/Browser/wwwtax.cgi?mode=Info&id=3352&lvl=3&lin=f&keep=1&srchmode=1&unlock) genome assembly version V1.01 was downloaded from NCBI (GenBank accession GCA_000404065.2 ). *Arabidopsis* thanina was downloaded from TAIR ([www.arabidopsis.org](http://www.arabidopsis.org/) version 10). The latest human genome sequence (version hg38) was retrieved from UCSC database (genome.ucsc.edu). Genome sequence assembly (v 1.01) of *Pinus taeda* was downloaded from tree gene database at <http://dendrome.ucdavis.edu/treegenes>. Genome sequence assembly of grasses was downloaded from public databases and the information were listed in Supplementary Table 1.

## Example statistical file output data from GMATA

Example data shows statistical information of SSR at five classifications in Zea Mays genome. Only the data in first several lines and last three lines were shown as an example here. Each statistical classification represents a different statistical view of SSR at whole genome aspects. SSR (monomer, minimum repeated times 12, and unit length 2-10 bp, minimum repeated times 12) in maize genome sequence was analyzed in GMATA using default setting. Statistical plotting was conducted used default settings.

| A   | Motif(-mer) | Total | Percentage | | --- | --- | --- | | 2 | 159030 | 54.28793806 | | 1 | 85944 | 29.33863138 | | 3 | 42862 | 14.63176508 | | 4 | 3318 | 1.132662884 | | 5 | 1070 | 0.365265005 | | 6 | 616 | 0.210283405 | | 7 | 95 | 0.032430071 | | 10 | 2 | 0.000682738 | | 8 | 1 | 0.000341369 | | total_above | total_above | total_above | | 9 | 292938 | 100 | | B   | Motif | Total | Percentage | | --- | --- | --- | | AT | 24620 | 8.4045088 | | TA | 23190 | 7.9163509 | | G | 23116 | 7.89108958 | | C | 22607 | 7.71733268 | | GA | 21086 | 7.19811018 | | CT | 20253 | 6.91374967 | | … |  |  | | GGTTT/AAACC | 1 | 0.00034137 | | AAAAGG/CCTTTT | 1 | 0.00034137 | | total_above | total_above | total_above | | 752 | 292938 | 100 | |
| --- | --- | --- | --- | --- | --- | --- | --- | --- | --- | --- | --- | --- | --- | --- | --- | --- | --- | --- | --- | --- | --- | --- | --- | --- | --- | --- | --- | --- | --- | --- | --- | --- | --- | --- | --- | --- | --- | --- | --- | --- | --- | --- | --- | --- | --- | --- | --- | --- | --- | --- | --- | --- | --- | --- | --- | --- | --- | --- | --- | --- | --- | --- | --- | --- | --- | --- | --- | --- | --- | --- | --- | --- | --- |
| C   | Grouped_Motif | Total | Percentage | | --- | --- | --- | | G/C | 45723 | 15.60842226 | | A/T | 40221 | 13.73020912 | | GA/TC | 37777 | 12.89590289 | | CT/AG | 35813 | 12.22545385 | | AT/AT | 24620 | 8.404508804 | | GT/AC | 9080 | 3.099632004 | | CG/CG | 7922 | 2.704326513 | | GC/GC | 7730 | 2.638783633 | | CAG/CTG | 6101 | 2.082693266 | | GCC/GGC | 2306 | 0.787197291 | | … |  |  | | AAAAGG/CCTTTT | 1 | 0.000341369 | | total_above | total_above | total_above | | 752 | 292938 | 100 | | E   | SSR_loci_length | Total_loci | Percentage | | --- | --- | --- | | 10 | 131769 | 44.9818733 | | 12 | 35910 | 12.25856666 | | 15 | 30638 | 10.45886843 | | 14 | 17415 | 5.944943981 | | 11 | 17080 | 5.830585312 | | 18 | 13926 | 4.75390697 | | … |  |  | | 141 | 1 | 0.000341369 | | 248 | 1 | 0.000341369 | | total_above | total_above | total_above | | 170 | 292938 | 100 | |  |  |  | |
| D   | SeqID | SSR_loci | Loci_percentage | SeqSize | Frequency(SSRs/Mb) | | --- | --- | --- | --- | --- | | >1 | 43192 | 14.7444169 | 3.01E+08 | 143.268 | | >2 | 34095 | 11.6389816 | 2.38E+08 | 143.306 | | >4 | 33507 | 11.4382566 | 2.42E+08 | 138.4231 | | >3 | 32679 | 11.1556029 | 2.32E+08 | 140.7088 | | >5 | 30624 | 10.4540893 | 2.18E+08 | 140.5032 | | >7 | 24908 | 8.50282312 | 1.77E+08 | 140.8614 | | >8 | 24835 | 8.47790317 | 1.75E+08 | 141.6088 | | >6 | 24617 | 8.4034847 | 1.69E+08 | 145.3121 | | >9 | 22067 | 7.53299333 | 1.57E+08 | 140.5201 | | >10 | 21411 | 7.30905516 | 1.5E+08 | 143.0909 | | … |  |  |  |  | | >scaffold_432 | 1 | 0.00034137 | 3288 | 304.1363 | | total_above | total_above | total_above | total_above | average_frequency | | 137 | 292938 | 100 | 2.07E+09 | 141.692 | | |

## Example marker design output by GMATA

The following data is an example data showing the marker designing output by GMATA after running the marker designing module using default setting.

1. File content and format in .mk file.

| SequenceID | MarkerID | PRIMER_LEFT_SEQUENCE | PRIMER_LEFT_TM | PRIMER_RIGHT_SEQUENCE | PRIMER_RIGHT_TM | LEFT_PRIMER_POS | RIGHT_PRIMER_POS | PRODUCT_SIZE |
| --- | --- | --- | --- | --- | --- | --- | --- | --- |
| >scaffold32|1:432|402:421 |  |  |  |  |  |  |  |  |
| >scaffold73|1:362|211:232 |  |  |  |  |  |  |  |  |
| >scaffold33|1:1281|79:100 | >MK1 | TGGGTTTGATTGAGTTTCTGG | 59.956 | GCTTCTTGCTGTGCTGAGTG | 59.929 | 28 | 313 | 286 |
| >scaffold132|1:403|86:99 | >MK2 | GGTGAACTGGCACAGAAGTG | 59.31 | TCAGTTGCGGCTAAAGGAAT | 59.845 | 26 | 362 | 337 |
| >scaffold148|1:459|118:133 | >MK3 | CAACCGGGAGTCGGTCTA | 59.625 | GTCGGGGTTGACGTAGTTGT | 59.891 | 78 | 310 | 233 |
| >scaffold190|1:387|336:345 |  |  |  |  |  |  |  |  |
| >scaffold214|1:191|72:81 | >MK4 | CGATCGAGTGACGTGATGA | 59.313 | TGTTCGGTGGCAACCAGT | 61.171 | 40 | 186 | 147 |
| >scaffold425|1:418|252:261 | >MK5 | GGGCATGTGAAGGGAAACTA | 59.933 | GGCTCCATGAGAATGTGGAT | 59.893 | 69 | 382 | 314 |
| >scaffold467|1:1168|582:600 | >MK6 | TCATCTCCCCAACAAACTCC | 59.903 | TTTGGTTCCTGGCCTAATTG | 59.931 | 465 | 735 | 271 |
| ... |  |  |  |  |  |  |  |  |
| >C254871006|1428:2242|1828:1842 | >MK123471 | CTGTCGAAGGAAGCCAAGAC | 59.989 | CGAAATGGGAAACTCCAGAA | 60.044 | 82 | 506 | 425 |
| >C254871006|5812:6626|6212:6226 | >MK123472 | CCGAAGGCAAATACGAGAAA | 60.202 | TTACCACTTTGCCCAAGACC | 59.971 | 79 | 543 | 465 |
| >C254870816|4548:5359|4948:4959 | >MK123473 | AGTCGAAGAGGAGACGCAAA | 60.134 | ATCTCGAGCCGTCGAAGTAA | 59.978 | 269 | 639 | 371 |
| >C254870982|235:1050|635:650 | >MK123474 | TGGAGATTTGCACGATTTCA | 60.197 | GAAACCTGGCGATCGTCTTA | 60.214 | 285 | 662 | 378 |
| >C254870908|2752:3566|3152:3166 | >MK123475 | TGCGTTGTCTTCTTTGTTGC | 60.035 | AAGTTTGCAGCCTTCCAAGA | 59.993 | 247 | 614 | 368 |
| >C254870908|6314:6734|6714:6733 |  |  |  |  |  |  |  |  |
| >C254871042|657:1474|1057:1074 | >MK123476 | CAGCACGAGTCGGACATCTA | 60.008 | TTTTGTCTCGCTGTTGTTGC | 60.035 | 245 | 492 | 248 |
| >C254871030|5923:6734|6323:6334 | >MK123477 | ATTCGACCATCGATCCAAGA | 60.426 | ACCGAGAAACAAACGAAACG | 60.147 | 368 | 800 | 433 |
| >C254871108|7877:8388|8277:8291 | >MK123478 | TAAGATGGTTTCCGGCAGAG | 60.206 | TTCCTCTAGCGAAGGTCTCAA | 59.205 | 85 | 493 | 409 |
| >C254871108|7897:8388|8297:8311 | >MK123478 | TAAGATGGTTTCCGGCAGAG | 60.206 | TTCCTCTAGCGAAGGTCTCAA | 59.205 | 65 | 473 | 409 |

2. File content and format in .sts file.

| MarkerID | Left_primer_sequence | Right_primer_sequence | Product_size |
| --- | --- | --- | --- |
| >MK1 | TGGGTTTGATTGAGTTTCTGG | GCTTCTTGCTGTGCTGAGTG | 286 |
| >MK2 | GGTGAACTGGCACAGAAGTG | TCAGTTGCGGCTAAAGGAAT | 337 |
| >MK3 | CAACCGGGAGTCGGTCTA | GTCGGGGTTGACGTAGTTGT | 233 |
| >MK4 | CGATCGAGTGACGTGATGA | TGTTCGGTGGCAACCAGT | 147 |
| >MK5 | GGGCATGTGAAGGGAAACTA | GGCTCCATGAGAATGTGGAT | 314 |
| >MK6 | TCATCTCCCCAACAAACTCC | TTTGGTTCCTGGCCTAATTG | 271 |
| >MK7 | AAACCACTGCGACCATTACC | GCTGGGATGTGGAGTTTGTT | 398 |
| >MK8 | CTCAATTAGGCCAGGAACCA | GGCACCAAGTTGAACCAGAA | 179 |
| >MK9 | TGAGAAACAGAAGCCCCAGT | GAAGTTGGGTCGAAATACCG | 407 |
| >MK10 | AGCTGAGCATCTGACGTCCT | TTTCATCCAGAACGCAACAA | 246 |
| ... |  |  |  |
| >MK123469 | TTTGTTGCAAGCTTCTCGTG | CATTGTGCTCGAAGCGATTA | 326 |
| >MK123470 | GCAAGCCTGTGAAAGTAGCC | GCACGAGTTTGTTGGCTGTA | 241 |
| >MK123471 | CTGTCGAAGGAAGCCAAGAC | CGAAATGGGAAACTCCAGAA | 425 |
| >MK123472 | CCGAAGGCAAATACGAGAAA | TTACCACTTTGCCCAAGACC | 465 |
| >MK123473 | AGTCGAAGAGGAGACGCAAA | ATCTCGAGCCGTCGAAGTAA | 371 |
| >MK123474 | TGGAGATTTGCACGATTTCA | GAAACCTGGCGATCGTCTTA | 378 |
| >MK123475 | TGCGTTGTCTTCTTTGTTGC | AAGTTTGCAGCCTTCCAAGA | 368 |
| >MK123476 | CAGCACGAGTCGGACATCTA | TTTTGTCTCGCTGTTGTTGC | 248 |
| >MK123477 | ATTCGACCATCGATCCAAGA | ACCGAGAAACAAACGAAACG | 433 |
| >MK123478 | TAAGATGGTTTCCGGCAGAG | TTCCTCTAGCGAAGGTCTCAA | 409 |

3. Example data showing Summary output file .sat3 of primer and marker designing

total SSR loci are : 290546

total ssr loci and percentage with primer pair designed are: 136374, 46.937145925258%

total ssr loci and percentage without primer pair designed are : 154172, 53.062854074742%

total NO. of unique markers is : 123478

# Supplementary Figures and Tables

## 2.1 Supplementary Figures

| 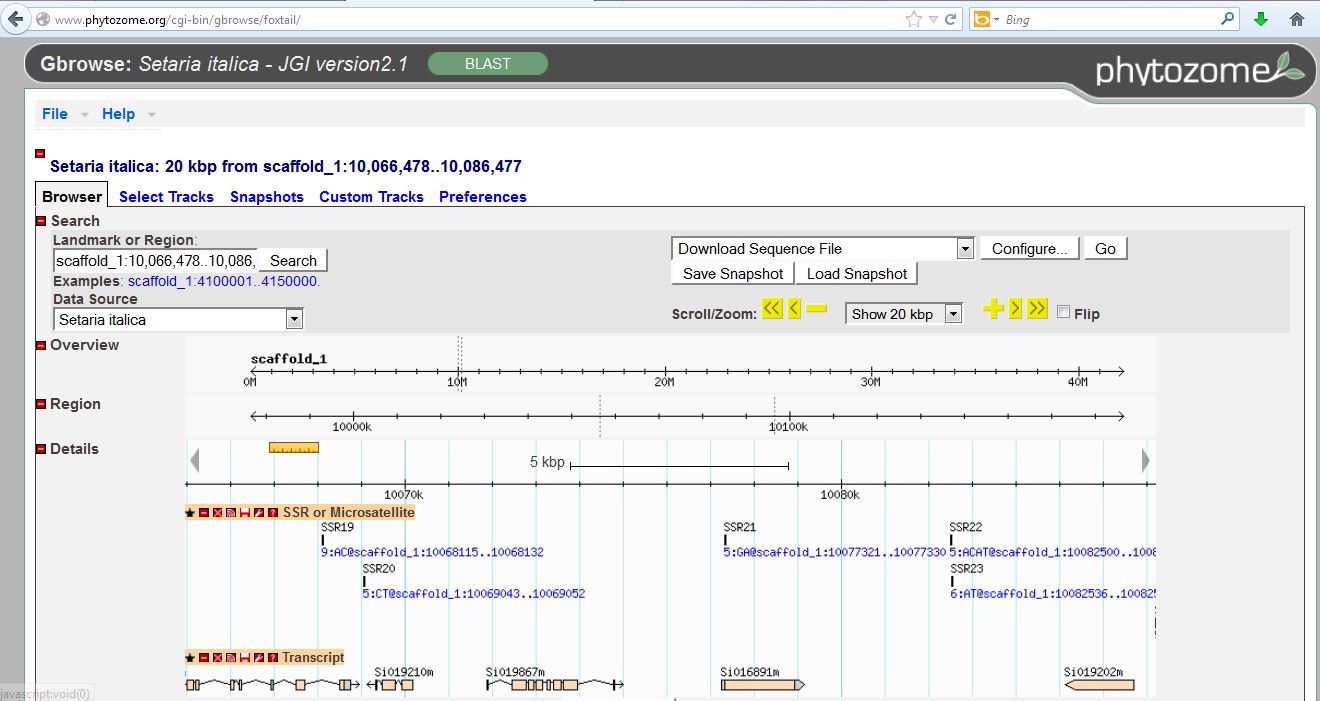 |
| --- |

## The display function of GMATA

SSRs (unit length 2-6 bp) from *Setaria* genome sequence were analyzed in GMATA using the default setting. The output from *Setaria* with the suffix .gbf was uploaded to the online Gbrowse browser at Phytozome and displayed with other genome features. Image showed SSR loci displaying the genome sequence and transcript information features

# 2.2 Supplementary Tables

## Genomic sequence sources of grass genomes

| Species | published year | Journal | Genome size | Sequence source | Genome assembly version | Transcript |
| --- | --- | --- | --- | --- | --- | --- |
| *Lolium perenne* | 2015.09 | the plant journal | 2 Gb | <http://185.45.23.197:5080/ryegrassgenome> | v1 | v1 |
| *Aegilops tauschii* | 2013.03 | Nature | 4.4 Gb | NCBI GenBank # GCA_000347335.1 | V1 | x |
| *Triticum urartu* | 2013.03 | Nature | 4.9 Gb | NCBI GenBank # GCA_000347455.1 | v1 | x |
| *Brachypodium distachyon* | 2010.02 | Nature | 260 Mb | phytozome | v3.0 | v3.1 |
| *Triticum aestivum(ABD, chromosome arm)* | 2014.07 | Science | 17 Gb | phytozome | v2.2 | *2.2* |
| *Triticum aestivum (ABD)* | 2012.11 | Nature | 17 Gb | EMBL ENA #CALP01000000 | v1 | x |
| *Setaria italica* | 2012.05 | Nature Biotechnology | 510 Mb | phytozome | v2 | v2.2 |
| *Hordeum vulgare* | 2012.01 | Nature | 5.1 Gb | EMBL ENA # CAJW01000000 | v1 | x |
| *Zea mays* ssp. *mays* | 2009.11 | Science | 2.3 Gb | phytozome | V3 | 5b+ |
| *Sorghum bicolor* | 2009.01 | Nature | 730 Mb | phytozome | V3.0 | v3.1 |
| *Oryza brachyantha* | 2013.03 | Nature Communications | 342 Mb | NCBI GenBank # GCA_000147395.2 | v1.4 | NCBI RefSeq # GCF_000231095.1 |
| *Oryza glaberrima* | 2014.08 | Nature genetics | 316 Mb | NCBI GenBank # GCA_000147395.2 | v1 | x |
| *Oryza sativa.* ssp. *japonica* | 2002.04 | Science | 466 Mb | phytozome | V7.0 | v7.0 |
| *Zizania latifolia* | 2015.06 | the plant journal | 590 Mb | EMBL ENA # ASSH01000000 | v1 | x |
| *Phyllostachys heterocycla* | 2013.12 | Nature Genetics | 2.1 Gb | <http://www.ncgr.ac.cn/bamboo> | v1.0 | v1 |

## Performance comparison of GMATA, SSRLocator and MISA software on whole genome sequence

|  | *Setaria Italica* genome (~500Mb) | | | | | | *Zea Mays* (2.1Gb) | | | | |
| --- | --- | --- | --- | --- | --- | --- | --- | --- | --- | --- | --- |
| Software | Time | | | SSR Loci | Loci with Primers | Markers | Time | Maxi Memory | SSR Loci | Loci with Primers | Markers |
|  | Windows* | Linux^#^ | Mac^&^ |  |  |  | Linux# |  |  |  |  |
| GMATA | 10m0s | 8m40s | 8m6s | 46,739 | 45,976 (3m22sec) | 41,277 | 41m59s | 598M | 206,705 | 203,178  (19m40s, maxi 813M) | 155,100 |
| SSR Locator | >12h^+^ | x | x | 46,625 | x | x | x | x | x | x | x |
| MISA | 16m11s | 12m14s | 15m13s | 46,782 | x | x | 78m45s | 897M | 206,820 | x | x |

For all tools, SSR motif length was set to 2 to 10 bp, minimum repeated times at least 5. SSR locator V1.1 and MISA was downloaded from official site [http://www.ufpel.tche.br/](http://www.ufpel.tche.br/faem/fitotecnia/fitomelhoramento/faleconosco.html) and <http://pgrc.ipk-gatersleben.de/misa/> respectively. Whole genome sequences of Sitalica_164.fa for foxtail millet (*Setaria Italica*) and Zmays_181.fa for Zea maize were downloaded from phytozome <http://www.phytozome.net/>.

* environment: HP 8000 Elite 32 byte Windows 7，Inter core2 CPU 2.83 GHz, 4G RAM，disk space 500 G； # environment: Linux gridview 2.6.18, 64 byte, AMD Opteron Processor 612 CPU 2.0 GHz, 66G RAM，disk space 500G; & environment: Mac Pro OS 10.7.5, Intel Xeon CPU 2.66 GHZ, 12G RAM，disk space 1T; + Summary of SSR loci was produced at 29m49s but it took more than 12 hours to export results of mined SSR data.

## Distribution of SSR identified by GMATA in fifteen grass genomes

| Species | published year | Journal | Genome size | Sequence source | Genome assembly version | Transcript |
| --- | --- | --- | --- | --- | --- | --- |
| *Lolium perenne* | 2015.09 | the plant journal | 2 Gb | <http://185.45.23.197:5080/ryegrassgenome> | v1 | v1 |
| *Aegilops tauschii* | 2013.03 | Nature | 4.4 Gb | NCBI GenBank # GCA_000347335.1 | V1 | x |
| *Triticum urartu* | 2013.03 | Nature | 4.9 Gb | NCBI GenBank # GCA_000347455.1 | v1 | x |
| *Brachypodium distachyon* | 2010.02 | Nature | 260 Mb | phytozome | v3.0 | v3.1 |
| *Triticum aestivum(ABD, chromosome arm)* | 2014.07 | Science | 17 Gb | phytozome | v2.2 | *2.2* |
| *Triticum aestivum (ABD)* | 2012.11 | Nature | 17 Gb | EMBL ENA #CALP01000000 | v1 | x |
| *Setaria italica* | 2012.05 | Nature Biotechnology | 510 Mb | phytozome | v2 | v2.2 |
| *Hordeum vulgare* | 2012.01 | Nature | 5.1 Gb | EMBL ENA # CAJW01000000 | v1 | x |
| *Zea mays* ssp. *mays* | 2009.11 | Science | 2.3 Gb | phytozome | V3 | 5b+ |
| *Sorghum bicolor* | 2009.01 | Nature | 730 Mb | phytozome | V3.0 | v3.1 |
| *Oryza brachyantha* | 2013.03 | Nature Communications | 342 Mb | NCBI GenBank # GCA_000147395.2 | v1.4 | NCBI RefSeq # GCF_000231095.1 |
| *Oryza glaberrima* | 2014.08 | Nature genetics | 316 Mb | NCBI GenBank # GCA_000147395.2 | v1 | x |
| *Oryza sativa.* ssp. *japonica* | 2002.04 | Science | 466 Mb | phytozome | V7.0 | v7.0 |
| *Zizania latifolia* | 2015.06 | the plant journal | 590 Mb | EMBL ENA # ASSH01000000 | v1 | x |
| *Phyllostachys heterocycla* | 2013.12 | Nature Genetics | 2.1 Gb | <http://www.ncgr.ac.cn/bamboo> | v1.0 | v1 |

Continued:


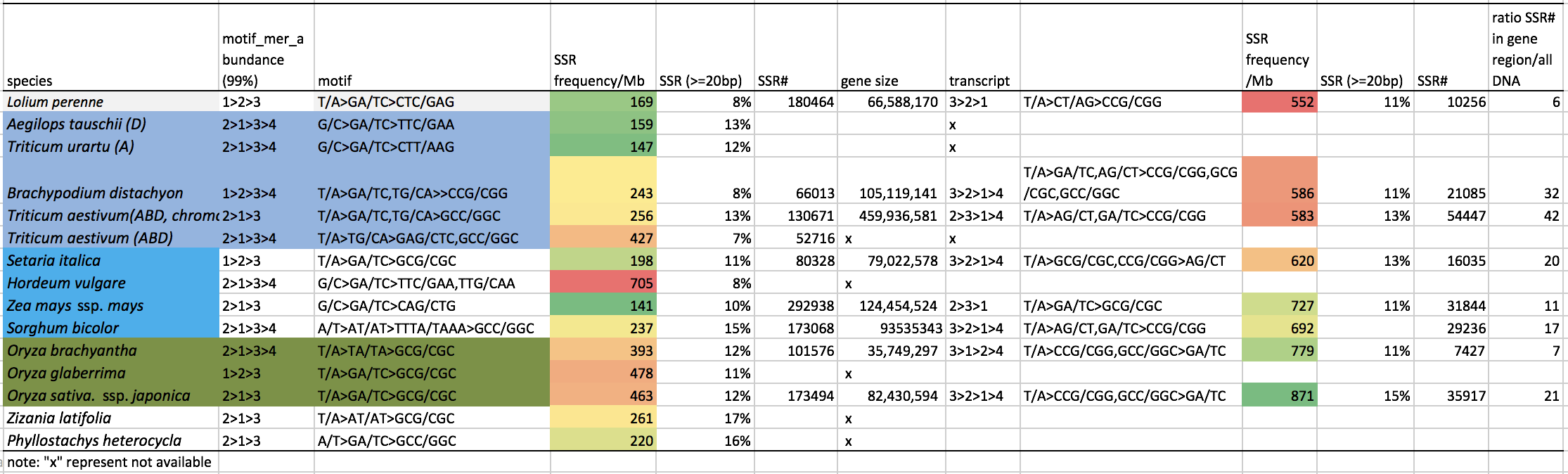

Supplement: Supplementary file 1 [file DataSheet1.DOC]
